# Characterization of Al_2_O_3_ Samples and NiAl–Al_2_O_3_ Composite Consolidated by Pulse Plasma Sintering

**DOI:** 10.3390/ma14123398

**Published:** 2021-06-19

**Authors:** Katarzyna Konopka, Marek Krasnowski, Justyna Zygmuntowicz, Konrad Cymerman, Marcin Wachowski, Paulina Piotrkiewicz

**Affiliations:** 1 Faculty of Materials Science and Engineering, Warsaw University of Technology, 141 Woloska St., 02-507 Warsaw, Poland; Katarzyna.Konopka@pw.edu.pl (K.K.); marek.krasnowski@pw.edu.pl (M.K.); konrad.cymerman.dokt@pw.edu.pl (K.C.); paulina.piotrkiewicz.dokt@pw.edu.pl (P.P.); 2 Faculty of Mechanical Engineering, Military University of Technology, 2 gen. S. Kaliskiego St., 00-908 Warsaw, Poland; marcin.wachowski@wat.edu.pl

**Keywords:** pulse plasma sintering, NiAl–Al_2_O_3_, composites, Al_2_O_3_ samples

## Abstract

The paper describes an investigation of Al_2_O_3_ samples and NiAl–Al_2_O_3_ composites consolidated by pulse plasma sintering (PPS). In the experiment, several methods were used to determine the properties and microstructure of the raw Al_2_O_3_ powder, NiAl–Al_2_O_3_ powder after mechanical alloying, and samples obtained via the PPS. The microstructural investigation of the alumina and composite properties involves scanning electron microscopy (SEM) analysis and X-ray diffraction (XRD). The relative densities were investigated with helium pycnometer and Archimedes method measurements. Microhardness analysis with fracture toughness (K_IC_) measures was applied to estimate the mechanical properties of the investigated materials. Using the PPS technique allows the production of bulk Al_2_O_3_ samples and intermetallic ceramic composites from the NiAl–Al_2_O_3_ system. To produce by PPS method the NiAl–Al_2_O_3_ bulk materials initially, the composite powder NiAl–Al_2_O_3_ was obtained by mechanical alloying. As initial powders, Ni, Al, and Al_2_O_3_ were used. After the PPS process, the final composite materials consist of two phases: Al_2_O_3_ located within the NiAl matrix. The intermetallic ceramic composites have relative densities: for composites with 10 wt.% Al_2_O_3_ 97.9% and samples containing 20 wt.% Al_2_O_3_ close to 100%. The hardness of both composites is equal to 5.8 GPa. Moreover, after PPS consolidation, NiAl–Al_2_O_3_ composites were characterized by high plasticity. The presented results are promising for the subsequent study of consolidation composite NiAl–Al_2_O_3_ powder with various initial contributions of ceramics (Al_2_O_3_) and a mixture of intermetallic–ceramic composite powders with the addition of ceramics to fabricate composites with complex microstructures and properties. In composites with complex microstructures that belong to the new class of composites, in particular, the synergistic effect of various mechanisms of improving the fracture toughness will be operated.

## 1. Introduction

Ceramic matrix composites are an important group of composites developed over many years. Ceramic–metal composites belong to this group of materials. New methods of fabrication and new types of these materials have been elaborated on. Metals such as Mo, V, Al, Ni, Cu, or Ti are often introduced into the ceramic matrix [1,2,3,4,5,6,7]. Metal particles located in the ceramic matrix interact with propagating cracks and cause deflection of the crack, bridging or stopping the cracks. As a consequence, the increasing fracture toughness of the composites has been observed [8,9,10,11,12]. However, it is not the only metal that is so active in improving the fracture behavior of brittle ceramic or intermetallic matrix composites. Intermetallic phases and other compounds are also regarded as reinforcement of composites [13,14,15,16,17,18,19]. Particular attention has been paid to intermetallic matrix composite particle-reinforced intermetallic compounds or ceramics. For example, in work [18], Al_2_O_3_ particle reinforced TiAl composites were reaction-synthesized from a powder mixture of Ti, Al, TiO_2_, and Nb_2_O_5_ by the hot-pressing method. In the TiAl matrix, Ti_3_Al, NbAl_3_ phases, and fine Al_2_O_3_ particles were found. The composites’ bending strength and fracture toughness reach the values of 398.5 MPa and 6.99 MPa m^1/2^, respectively [18]. Other examples of investigation of composites of the Al_2_O_3_-TiAl system can be found, for example, in papers [16,17,19].

Another interesting system is Al_2_O_3_–NiAl [20,21,22] or NiAl/TiC–Al_2_O_3_ [23]. Combining these ceramics with intermetallic phases allows producing composites for high-temperature and chemical-resistant applications. Moreover, the introduction of the intermetallic phase into the ceramic matrix and ceramics into the intermetallic matrix can efficiently improve the fracture toughness of composite materials of intermetallic–ceramics systems. Because of that, the NiAl–Al_2_O_3_ system has been intensively investigated, especially the composites in which NiAl is a matrix and alumina is a reinforcing phase [22,24,25,26,27]. Abe and Ohwa, in their work [27] prepared NiAl–Al_2_O_3_ composites by the pressureless sintering of the powders synthesized via chemical precipitation route. The dominant formation of NiAl_2_O_4_ was observed in composites. The compressive stress for an oxidized NiAl–Al_2_O_3_ composite was equal to 127 MPa, and the improved fracture toughness was equal to 6.2 MPa m^1/2^ [27].

Primarily, the reinforcing phase is prepared separately before the composite fabrication. Then the consolidation of mixed powders is provided to produce bulk composite materials. For example, at work [25], dense Al_2_O_3_-NiAl composites were prepared by hot pressing. Al_2_O_3_ and NiAl powders were used as starting powders. However, ceramic matrix and intermetallic matrix composites can also be achieved not by consolidating the blended powders of matrix and reinforcement but by consolidating previously prepared composite powders.

There are different methods for producing composite powders. In methods called in situ, compounds are created by chemical reactions. As a result, very fine reinforcement particles and their homogeneity distribution in the matrix can be obtained in the composite powder. For example, Beyhaghi et al. [22,24] produced nanocomposite powders NiAl–Al_2_O_3_ via the mechano-synthesis route. As initial substrates, Ni, NiO, and Al powders were used. As a result of in situ synthesis, nano-crystallites of NiAl and Al_2_O_3_ were obtained. In another work [21], NiAl–Al_2_O_3_ composites were sintered in-situ from Al powder and preoxidized Ni particles by aluminothermic reactions. Mechanical alloying (MA) is a popular method of synthesis materials [28,29,30,31]. This method successfully produces intermetallics and intermetallic matrix composites [28,32,33,34]. There are other techniques of intermetallics synthesis, such as other methods of powder metallurgy, self-propagating high-temperature synthesis (SHS), or rapid solidification [35,36,37]. However, the synthesis of intermetallic materials requires special conditions because of the restrictive stoichiometry of this compound and the complex crystal structure. In mechanical alloying, high-energy ball milling is involved in the synthesis of the intermetallic material and, during this, the chemical reaction and phase transformation have been complied with. The parameters of the mechanical alloying process and used reactants control the final product.

In the present paper, NiAl–Al_2_O_3_ composite powder was produced and then consolidated. To form the intermetallic phase, Ni and Al powders were used. The process of milling (MA) began with metals and added ceramic powder (Al_2_O_3_). This allowed us to obtain a uniform distribution of constituencies in the composite powder and then keeping it in the bulk composite obtained by consolidating them. During the MA process, the Ni with Al will constitute the NiAl with trapped inside Al_2_O_3_ particles. After the consolidation of such composite powder in bulk material, it should be the positive result of improving the fracture toughness. Significantly, the redirecting of crack propagation by Al_2_O_3_ particles was expected. Moreover, the composite powder NiAl–Al_2_O_3_ can also be mixed with ceramic powder and consolidated to obtain the final ceramic–intermetallic bulk composite. The microstructure of such forming composite will be complex and consists of a ceramic matrix distributed in its intermetallic phase with ceramic particles trapped inside. Such a method of producing the ceramic matrix composites with the contribution of intermetallic phase with the ceramic particles inside is not commonly presented in the literature. In the scope of own research, such composites are included. However, initially, the composite NiAl–Al_2_O_3_ powder fabrication by the proposed MA method must be elaborated and characterized and the following consolidation process. As a method of composite consolidation, pulse plasma sintering (PPS) was selected.

In this technique, the material was heated by electric pulses generated periodically by a discharged capacitor battery and, at the same time, subjected to uniaxial pressing. The application of a capacitor battery as a source of electrical energy allows the electric pulses to be produced periodically over several hundred microseconds and a current intensity of about 100 kA [38,39]. Through the short duration of the electric pulse related to the time interval between the individual pulses, the temperature achieved during the pulse is higher than that stabilized during the traditional sintering method [38,39]. The temperature of the specimen and its heating rate are regulated by controlling the energy dissipated through the electric pulses, including adjusting the intervals between consecutive pulses. The choice of this method was based upon the advantage of short sintering time, which gives the possibility to rapidly sinter bulk materials and avoid the crystal coarsening [39]. In the experimental work, Al_2_O_3_ powder was also consolidated by the PPS method. This research aims to recognize the PPS method as a possible technique to produce bulk composite materials from ceramic powder and a mixture of composite powder NiAl–Al_2_O_3_.

The characterization of pure Al_2_O_3_ powder and NiAl–Al_2_O_3_ composite powder before and after the PPS consolidation is presented. These experiments will be treated as the beginning of further research on the fabrication of ceramic–intermetallic composites using composite NiAl–Al_2_O_3_ powder.

## 2. Materials and Methods

### 2.1. Initial Powders and Preparing the Composite Powder

In this experiment, the following powders were used: α-Al_2_O_3_ powder (MARTOXID^®^ MR-5, Martinswerk GmbH, Bergheim, German) with an average particle size range of 0.3–6 µm, Ni powder (ABCR GmbH and Co.KG, Karlsruhe, German) with an average particle size ranged from 3 µm to 7 µm and Al powder (ABCR GmbH and Co.KG, Karlsruhe, German) with average particle size equal 44 µm. Characterization of the raw powders was performed based on data contributed by the manufacturer. The powders used for milling were Ni (99.9% purity), Al (99.7% purity), and Al_2_O_3_ (99.98% purity).

The first stage of the research was producing composite powders based on Al_2_O_3_, Ni, and Al due to mechanical alloying. For the milling processes we used powder blends of Ni-50at.%Al with the addition of 10 wt.% and 20 wt.% of Al_2_O_3_. The milling processes were carried out in a high-energy SPEX 8000 D shaker ball mill (SPEX^®^ SamplePrep, Metuchen, NJ, USA). The ball-to-powder weight ratio was 10:1. The milling processes and sampling of powders were conducted under the protective atmosphere of Ar.

### 2.2. Pulse Plasma Sintering (PPS) Process

**Figure 1:**
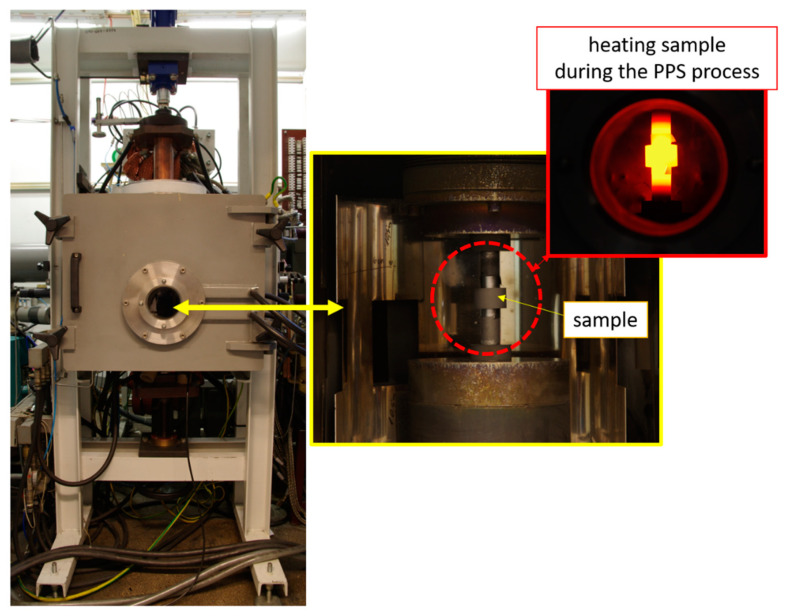
View of equipment use to produce composites by PPS.

The PPS method was used to produce the bulk specimens from the prepared composite powder. Table 1 gives the PPS process parameters. In Figure 1, the equipment used to produce composites by the PPS method is shown. The powder is loaded into the matrix and heating by the heat generated during the electric pulses. After the process, the disc samples were obtained. In the experiment, samples containing 100% Al_2_O_3_ were sintered at various temperatures (1000 °C, 1100 °C, 1200 °C, 1300 °C, 1400 °C, 1500 °C) in order to choose the proper temperature for sintering. The following temperatures in the PPS process were determined base on the required temperature of Al_2_O_3_ sintering, which is generally estimated with melting temperature and is close to 1450 °C.

On the other hand, as mentioned in the Introduction, the temperature of consolidation achieved in the PPS process is higher than in the traditional sintering method [38,39]. Because of that, 1500 °C was the higher applied temperature of the process. Moreover, to control the progress of sintering in the PPS method, experiments were undertaken with the proposed range of temperature, starting at 1000 °C.

**Figure 2:**
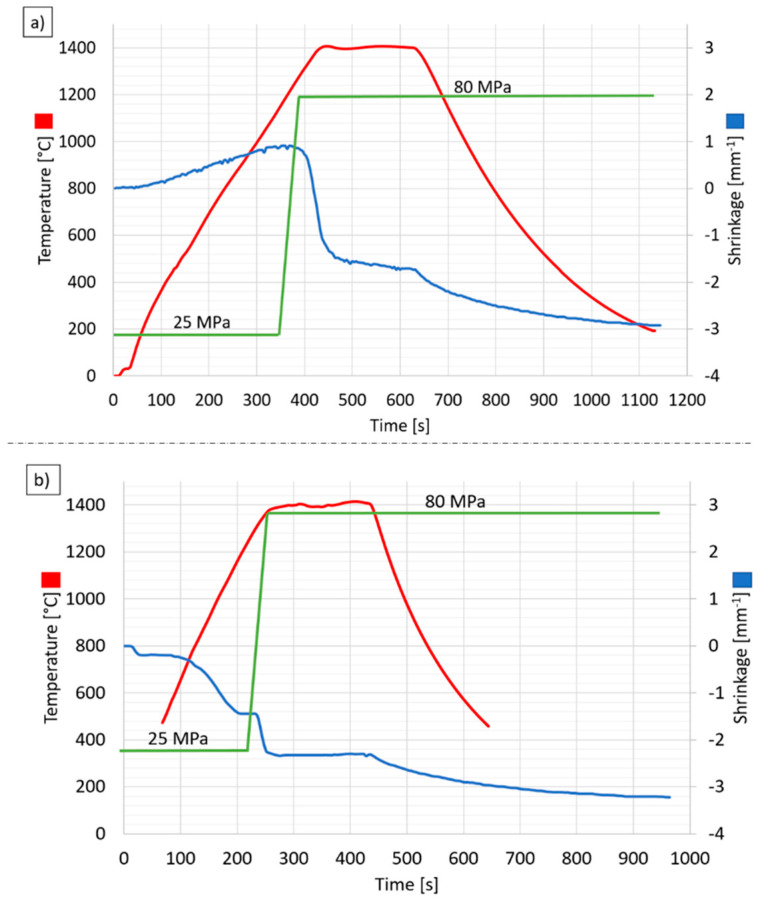
Graphs of temperature, shrinkage speed, and pressure during sample sintering at 1400 °C: (**a**) Al_2_O_3_ sample, (**b**) composite sample.

An exemplary record of changes in temperature, shrinkage rate, and pressing pressure during the PPS sintering process for the Al_2_O_3_ sample and composite sample is presented in Figure 2. The displacement of the stamp measured by a laser extensometer is used to estimate the consolidation progress, which is called shrinkage.

Based on the analysis of Figure 2a, it can be concluded that for the Al_2_O_3_ powder during the PPS process at the temperature of about 1200 °C, the powder only expands. In this temperature range (20–1200 °C), no shrinkage of Al_2_O_3_ powder was observed. Above the temperature of 1200 °C, sample shrinkage begins, i.e., the consolidation stage during PPS. At the same time, when the start of shrinkage of the sample, the pressure was increased to 80 MPa (green line in the diagram). Based on the graph obtained, it can be concluded that during 3 min of being at the target temperature (1400 °C), the Al_2_O_3_ sample is finally consolidated. This is confirmed by a slight shrinkage visible in the chart (Figure 2a—blue line). In NiAl–Al_2_O_3_ powder, it was found to shrink after the first pulse (Figure 2b). The obtained diagram found that more significant NiAl–Al_2_O_3_ powder shrinkage occurs after exceeding 500 °C and lasts up to 1200 °C (Figure 2b—blue line). Another shrinkage observed at 1300 °C is related to the increase in pressure to 80 MPa. At the target temperature, which is 1400 °C, no shrinkage of the NiAl–Al_2_O_3_ sample was observed. The process flows for the samples produced by the PPS method presented in Figure 2 are representative. The shrinkage characteristics during the process shown in Figure 2a,b have been different. This is because, in the research, we used two other materials with different thermal expansion coefficients.

### 2.3. Experimental Techniques

In the experiment, several methods were used to determine the properties and microstructure of the raw powder, powder after mechanical alloying, and samples obtained via the PPS. The phase composition and the structure of the powders after different milling times and powders after consolidation by the PPS technique were investigated by X-ray diffraction (XRD). The examination was accomplished by a Rigaku Miniflex II X-ray diffractometer (Rigaku Corporation, Tokyo, Japan) with Cu Kα (λ = 1.54178 Å, 15 mA, 30 kV) at a step size 0.05° with radiation in the 2θ range from 23° to 123°. For assessing the mean crystallite size, the Williamson–Hall method was employed (the instrumental broadening was subtracted from the experimental breadth to obtain the physical broadening of each diffraction line).

To establish the level of sintering compaction, the relative density was determined using the density of the powders designated using a helium pycnometer (AccuPyc 1340 II by Micromeritics, Norcross, GA, USA). The Archimedes method was applied to calculate the apparent and relative density, open porosity, and absorptivity of samples prepared by the PPS technique. According to the European Standard ISO 18754:2013 (EN), Archimedes’ method was measurement [40].

The hardness of the prepared samples was measured by the Vickers method on the polished sample surface under a load of 20 kG with a 10-s holding time. The hardness tester HVS-30T (Huatec Group Corporation, Beijing, China) was used to determine the hardness. For each sample, at least 15 measurements were made. The corresponding indentation sizes were determined using diagonals, measured using a light microscope Nikon Eclipse LV15ON (Nikon Corporation, Tokyo, Japan). Based on the length of cracks propagating from the corner of the hardness indentation, the material’s fracture toughness (K_IC_) was determined. In this investigation, a Vickers hardness indenter was applied to propagate the median cracks on the surface.

Observations of the microstructure and morphology of source Al_2_O_3_, Ni, and Al powders, composite powder after mechanical alloying, and fabricated bulk samples were carried out using a JEOL JSM-6610 scanning electron microscope—SEM (JEOL, Tokyo, Japan). Before observation, the samples were carbon-coated using the Quorum Q150T ESS coating system. The observation was performed using a secondary electron detector (SE) and a back-scattered electron (BSE) detector. A voltage of 15 kV was applied during the observations. Surface microanalysis of the chemical composition was performed using an X-Max type energy-dispersive X-ray spectrometer (EDS, Hitachi High-Tech Corp., Oxford, UK) to determine the elemental distribution in the obtained powder particles of the MA products and composites after sintering via PPS.

The changes in the size distribution of the raw Al_2_O_3_ powder and Al_2_O_3_ grains in the bulk samples of pure alumina obtained at different temperatures were examined using a stereological analysis. A quantitative description of the microstructure of the specimens was carried out based on scanning electron microscopy (SEM) images of randomly chosen areas on the fracture of samples. The quantitative description was carried out using a MicroMeter v.086b computer image analyzer [41,42].

## 3. Results and Discussion

### 3.1. Initial Powder Characterization

**Figure 3:**
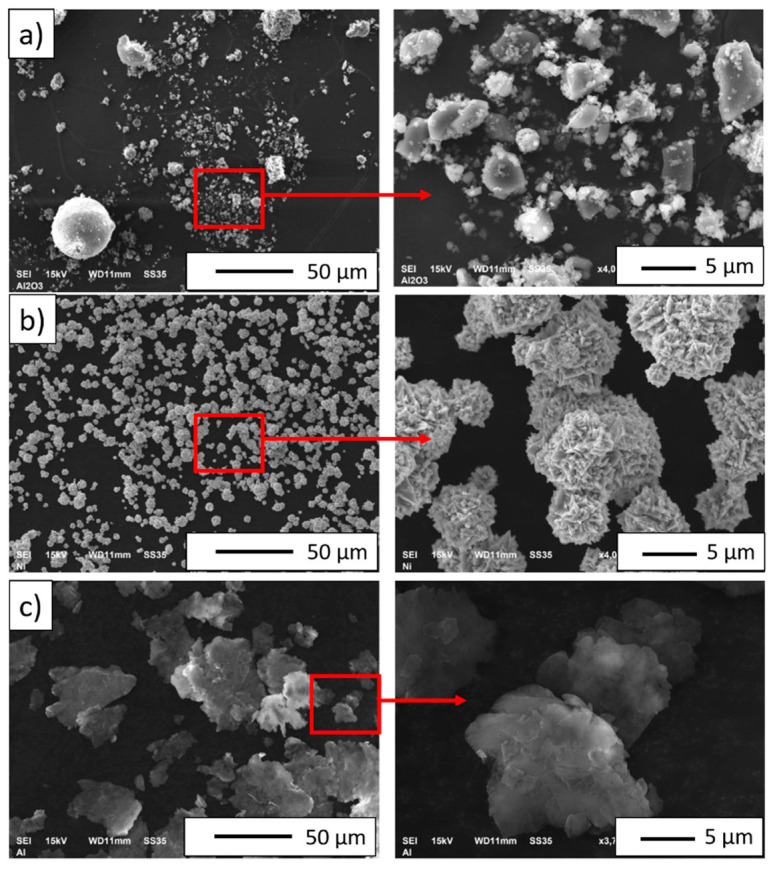
Scanning electron microscopy (SEM) images of the used powders: (**a**) aluminum oxide, (**b**) nickel, (**c**) aluminum.

Figure 3 shows the scanning electron microscopy images of the base Al_2_O_3_ and metallic powders. Analysis of SEM micrographs revealed significant diversification in the morphology of the starting powders. It was observed that aluminum oxide, nickel, and aluminum powders (Figure 3a–c) featured an irregular morphology. The Al_2_O_3_ powder has various shapes, oval, rectangular in cross-section, and irregular forms are visible (Figure 3a). Furthermore, the aluminum oxide powder tends to form agglomerates with high size distribution variation. The nickel powder has the shape of spherulite (Figure 3b). The Al powder has irregular surfaces (Figure 3c).

**Figure 4:**
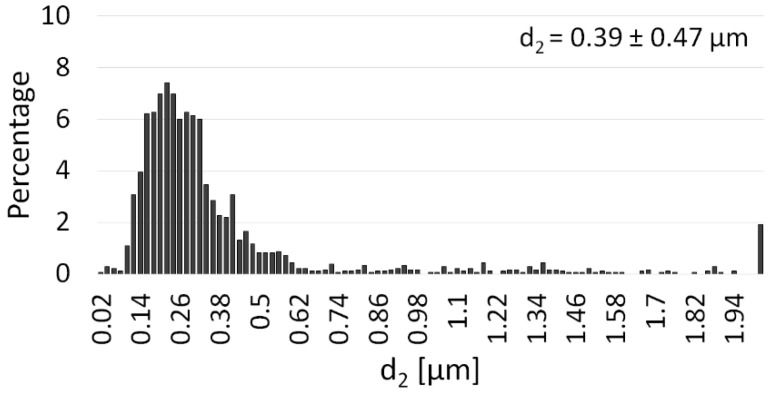
Histograms of the particle size of the Al_2_O_3_ powder.

Figure 4 shows a histogram of the particle size distribution of the Al_2_O_3_ powder. Based on the histogram, it can be assumed that the Al_2_O_3_ distribution is almost unimodal, with particles ranging from 0.02 µm to 5.30 µm in size. The results obtained showed that the average particle size of the alumina was about 0.39 µm. The results of the Al_2_O_3_ particle size distribution analysis are consistent with the data provided by the manufacturer.

The density of Al_2_O_3_ was equal to 3.93 g/cm^3,^ which corresponds to the value given by the manufacturer.

### 3.2. NiAl–Al_2_O_3_ Composite Powder Characterization

**Figure 5:**
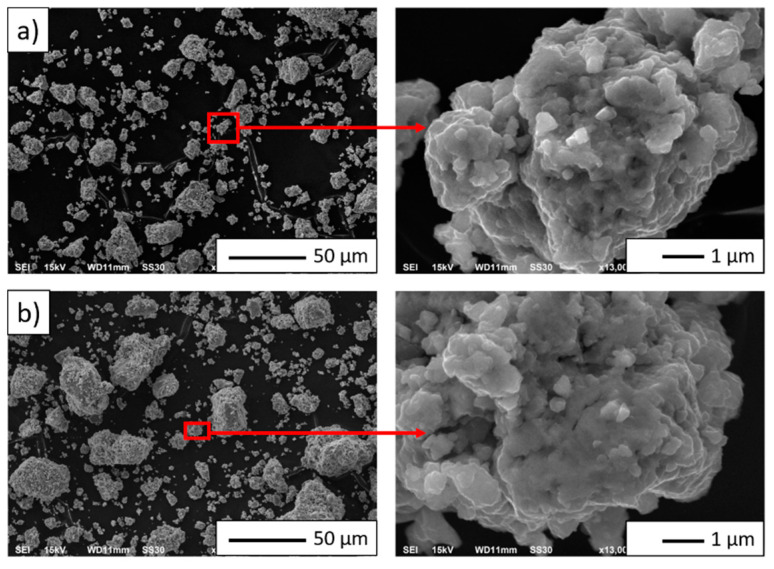
SEM images of the powder particles of the mechanical alloying (MA) product: (**a**) NiAl-10%Al_2_O_3_ powder, (**b**) NiAl-20%Al_2_O_3_ powder.

The powders’ density was determined, and for the NiAl-10%Al_2_O_3_ powder, it was 5.561 g/cm^3^, while for the NiAl-20%Al_2_O_3,_ it was equal to 5.236 g/cm^3^. As shown in Figure 5, the composite powders make spheroidal agglomerates. Fine (below 1 µm) as well as large agglomerates up to 50 µm were observed.

**Figure 6:**
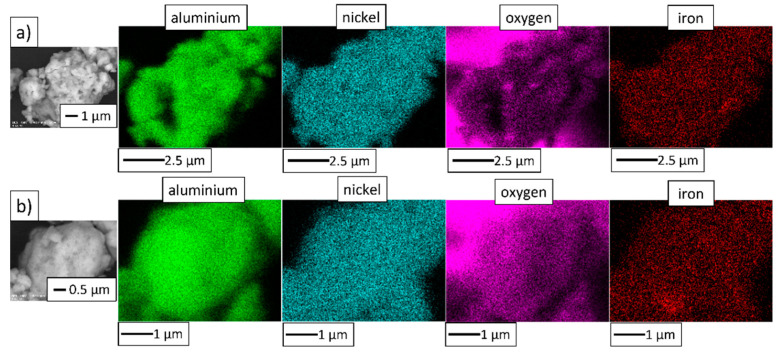
Elemental distribution maps of the powder particles of the MA product: (**a**) NiAl-10%Al_2_O_3_ powder, (**b**) NiAl-20%Al_2_O_3_ powder.

Figure 6 shows EDS maps of the MA product. Besides nickel, aluminum, and oxygen, the presence of iron was detected. Contamination of powders by Fe from steel milling tools is commonly observed in mechanical alloying processes [28].

**Figure 7:**
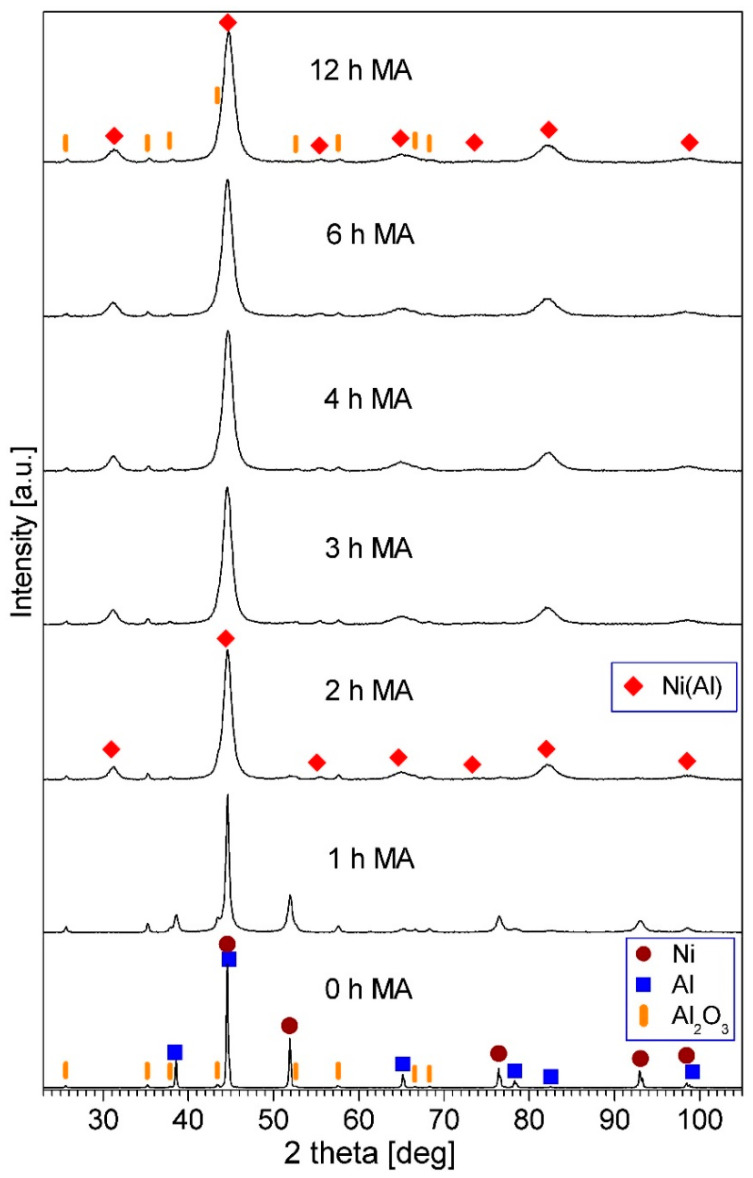
X-ray diffraction (XRD) patterns of the Ni-50at.%Al+10wt.%Al_2_O_3_ powder mixture milled for the times quoted.

Phase development in the Ni_50_Al_50_-Al_2_O_3_ powder mixtures during mechanical alloying can be examined based on the XRD patterns of the powders after various milling times. Figure 7 displays the patterns for the milled Ni-50at.%Al-10wt.%Al_2_O_3_ sample. It can be seen that after two h of mechanical alloying, a new phase was formed, which is demonstrated by the appearance of novel peaks in the XRD pattern. These peaks have been assigned to a NiAl phase. At the same time, the intensity of Ni and Al peaks decreased significantly. In the pattern for the three h-milled powder mixtures, all Al and Ni peaks vanished. This indicates that all Ni reacted with Al, creating a NiAl intermetallic phase, at least partially ordered. The observed reaction between Ni and Al and phase development during the formation of the NiAl phase was analogous to those described earlier for mechanical alloying of Ni-50at.%Al powder mixture performed in the same kind of mill [43]. The diffraction peaks of Al_2_O_3_ were present in the discussed XRD patterns all while. The phase composition of the powder mixture did not change for longer milling times. For the Ni-50at.%Al-20wt.%Al_2_O_3_ sample, the same phase evolution was observed.

It can be seen in Figure 7 that the NiAl diffraction peaks broadened with the milling time extension. This broadening was due to the reduction in the crystallite size of the NiAl phase and the increase in lattice microstrains in this phase [44]. Since some of the NiAl peaks overlap with the Al_2_O_3_ peaks, for the analysis of the peaks’ width and Williamson–Hall calculations the peaks were separated by fitting and deconvolution. The estimated mean crystallite size of the NiAl phase in the final milling product was 14 nm and 12 nm for the sample containing 10% and 20% of Al_2_O_3_, respectively.

The produced powders have a composite structure with Al_2_O_3_ particles distributed in the nanocrystalline NiAl intermetallic matrix. Interestingly, literature data show that Krasnowski et al. obtained a similar structure for NiAl-B powder [32]. Krasnowski et al. conducted the process of mechanical alloying for Ni, Al, and B powders. They managed to manufacture a powder with a composite structure, in which fine B particles were homogeneously distributed in a nanocrystalline NiAl matrix [32].

### 3.3. Characterization of Al_2_O_3_ Powder Compacted by PPS

The PPS consolidation method was firstly applied for pouring Al_2_O_3_ powder at the range of temperature from 1000 °C up to 1500 °C to describe the sintering process of ceramic powder in the function of temperature. The density, hardness, and size of ceramic grains in particular were examined in the function of sintering temperature. The results of these experiments were used to select a proper temperature of PPS consolidation for the composite NiAl_2_O_3_-Al_2_O_3_ powder. The crucial was to sinter the composite powder and not allow for grain growth of Al_2_O_3_ particles.

**Figure 8:**
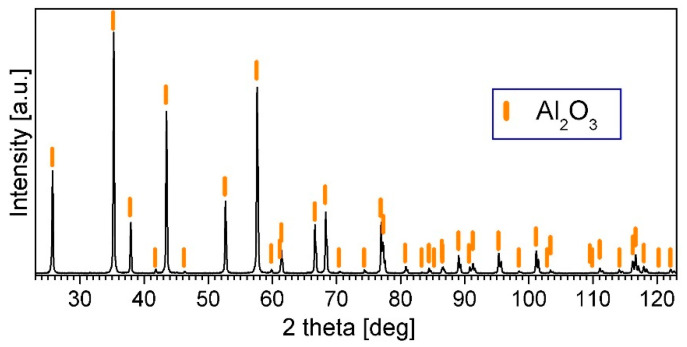
XRD pattern of the A_2_O_3_ powder after consolidation by PPS.

In the XRD patterns of the Al_2_O_3_ powder after consolidation by PPS at various temperatures, only the peaks of Al_2_O_3_ are visible. Figure 8 shows the pattern of the sample sintered at 1400 °C as an example.

The Vickers hardness measured for compacted samples was used to compare the results and estimate fracture toughness (Table 2). It was found that the Al_2_O_3_ composite samples sintered in the higher temperature (1500 °C) exhibited hardness equal to 15.3 ± 0.87 GPa. The hardness value determined for the samples sintered at 1400 °C amounted to 15.1 ± 0.8 GPa. It may be noted that the hardness values achieved for both samples are similar (values are in the range of measured errors). The relative density of both specimens remained relatively high. Samples sintered at 1400 °C were characterized by a relative density around 97% of the theoretical density, while samples sintered at 1500 °C presented a relative density close to 100% of the theoretical density. The measured values of densities for samples are shown in Table 3. The difference in density between the samples appeared to have no significant effect on the hardness results. For Al_2_O_3_ samples sintered at lower temperatures, the quality of the consolidation process was too poor, which made hardness testing impossible.

Vickers indentation fracture toughness measurements determined the fracture toughness (K_IC_—critical stress intensity factor). For a thorough comparative analysis, several equations, summarized in Table 2, have been used to calculate K_IC_ values. While the Niihara and Anstis equations apply to the median type of crack, the Lankford equation can be used for any kind of crack. Unified symbol designations were used in all equations applied to determine K_IC_ coefficient values. Therefore “E” corresponds to Young’s modulus, “HV” to hardness, and “F” to the load applied, “a” is half-length of the diagonal of the Vickers indentation, and through “c” the crack length from the center of the indentation to the crack tip is given.

**Figure 9:**
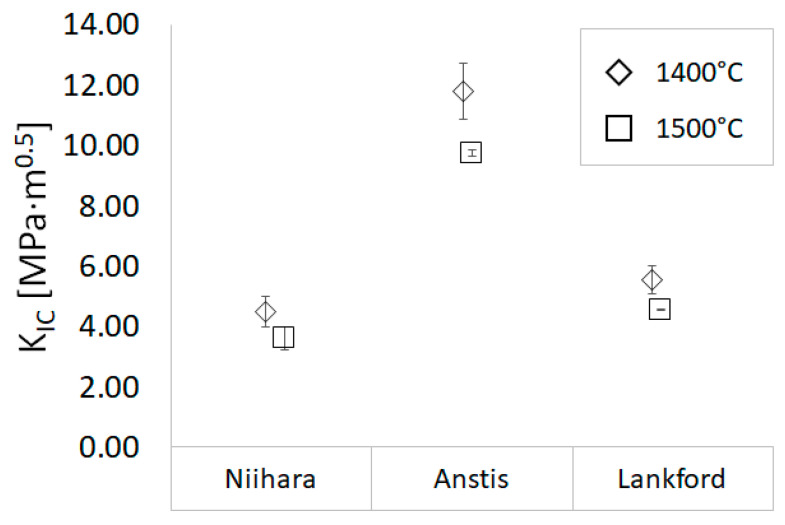
Fracture toughness of sintered Al_2_O_3_ samples prepared via PPS calculated by using different equations.

The results of the indentation fracture toughness analysis are presented in Figure 9. The critical stress intensity factor (K_IC_) values depend strongly on the equation applied for the calculations. However, it is noticeable that the general tendency among the K_IC_ values of the examined series remains constant. Regardless of the equation used for the accounting, higher fracture toughness was characterized by samples sintered at a lower temperature. The K_IC_ values for the specimen sintered at 1400 °C were in the range 4.53–11.81 MPa·m^0.5^, while the values for the sample sintered at 1500 °C varied from 3.65 to 9.78 MPa·m^0.5^.

Regarding samples made from Al_2_O_3_ ceramics, Maiti et al. [45], in their study, achieved comparable hardness values ranging from 9–13.2 GPa, depending on the sintering time. The samples were made by solid-state sintering after initial uniaxial pressing with polyvinyl alcohol (PVA). They were characterized by lower relative density values than the samples in this paper (96–98%) [45]. Hardness values similar to those presented in this study were also obtained by Ouyang et al., which were uniaxially pressed spheroidal Al_2_O_3_ powder sintered at 1550 °C. Depending on the holding time at the sintering temperature, the hardness of the samples was in the range of 16–18 GPa, with relative densities not exceeding 96% of the theoretical density [46].

In the case of literature reports on the hardness of Al_2_O_3_ ceramics prepared by the field-assisted sintering technique/spark plasma sintering (FAST/SPS), the hardness values obtained are significantly higher at similar densities. In the work of Xu et al., where Al_2_O_3_ was sintered with the current-assisted sintering (ACS) method, which is a modification of the classical spark plasma sintering (SPS), at a pressure of 30 MPa, the hardness values obtained at 1400 °C and 1500 °C were 19.24 GPa and 17.76 GPa, respectively [47]. Similar values were also brought in the work of Yuan et al., where oscillatory pressure sintering was used to obtain samples with densities above 95% and hardness in the range 17–23 GPa [48].

Due to the different methods for determining fracture toughness and, in the case of the Vickers indentation test, the high dependence of the results obtained on the choice of the equation, it is difficult to compare the data available in the literature regarding fracture toughness. However, it is worth mentioning that the fracture toughness determined in the work of Maiti et al. [45] with comparable hardness values was lower than in the present study. The value calculated with the Anstis equation was in the range 5.2–5.4 MPa·m^0.5^, while in the following work, the values of K_IC_ calculated in the same way reached the value above 10 MPa·m^0.5^. In the study of Žmak et al. [49], where solid Al_2_O_3_ samples were fabricated by slip casting and sintered at 1650 °C, the calculated K_IC_ values from the Niihara and Lankford equations remain in good correlation with the results derived in this paper, with K_IC_ values of 4.89 MPa·m^0.5^ and 5.29 MPa·m^0.5^, respectively. According to Chakravarty et al. [50], Al_2_O_3_ ceramic specimens were fabricated by the SPS method at 1300 °C, K_IC_ values ranging from 3.25 MPa·m^0.5^ to 3.45 MPa·m^0.5^ were derived based on the Chantikul equation. Belmonte et al. [51] used the single beam notch method to determine the K_IC_ values of Al_2_O_3_ ceramic samples sintered by hot-pressing at 1500 °C. The obtained K_IC_ values for Al_2_O_3_ amounted to 4.3 MPa·m^0.5^.

**Table 2:** Equations applied in the calculation of Vickers indentation fracture toughness.

Author	Equation	Type of Crack System
Niihara [52]	$K_{IC}=0.067\cdot {\left(\frac{E}{HV}\right)}^{0.4}\cdot {\left(\frac{c}{a}\right)}^{-1.5}\cdot \left(HV\cdot a^{0.5}\right)$	Radial—median
Anstis [53]	$K_{IC}=0.016{\left(\frac{E}{HV}\right)}^{0.5}\cdot \frac{F}{c^{1.5}}$	Radial—median
Lankford [54]	$K_{IC}=0.0782\cdot \left(HV\cdot a^{0.5}\right)\cdot {\left(\frac{E}{HV}\right)}^{0.4}\cdot {\left(\frac{c}{a}\right)}^{-1.56}$	Any kind

**Table 3:** The helium pycnometer measurements and Archimedes measurements of the Al_2_O_3_ samples at the range of temperature from 1000 °C up to 1500 °C.

Sintering Temperature	Results of the Helium Pycnometer Measurements and Archimedes Measurements				
	Density Determined by Helium Pycnometer [g/cm^3^]	Apparent Density [g/cm^3^]	Relative Density [%]	Open Porosity [%]	Water Absorptivity [%]
1000 °C	3.93	2.6168	66.58	31.50	12.04
1100 °C	3.93	2.7309	69.49	28.23	10.34
1200 °C	3.93	2.9364	74.72	24.29	8.27
1300 °C	3.93	3.2498	82.69	16.79	5.17
1400 °C	3.93	3.8088	96.92	0.81	0.21
1500 °C	3.93	3.9298	99.99	-	-

The results of the helium pycnometer measurements and Archimedes measurements were presented in Table 3. It was observed that with the increasing temperature of the sintering process, the relative density of the samples increased. Analysis of the results obtained based on the Archimedes method indicated that the lowest relative density 66.58% values were observed for samples prepared at the lowest temperature of sintering (1000 °C). The highest open porosity value also characterized the same sample among all examined ones. On the other hand, the highest values were observed for the samples prepared at 1500 °C. They were close to the total density (99.99%). The literature reports the results of PPS sintering of intermetallic and ceramics materials where very dense structures are obtained [55]. In our own (not published) preliminary experiments of ceramics consolidation by PPS, the high density of samples was noticed too.

Porosity and water absorption of the produced samples decreased with increasing density according to the well-known rule [56]. Table 3 does not show the results of open porosity and water absorption for the specimen sintered at 1500 °C because this material had open porosity close to zero and consequently did not exhibit any water absorption.

**Figure 10:**
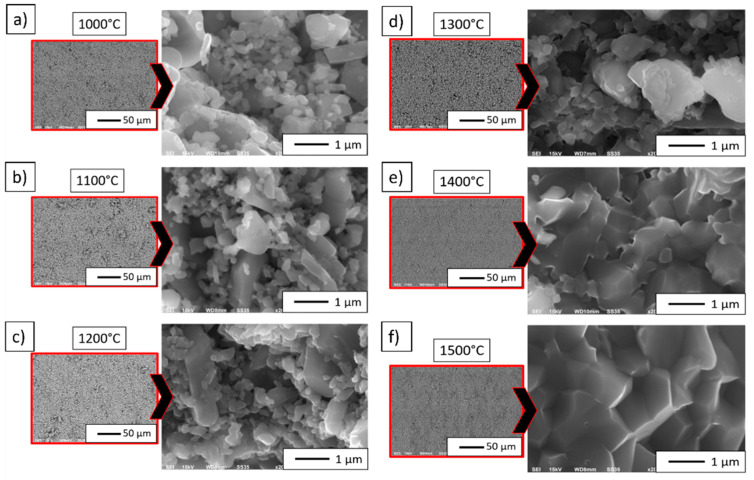
Fracture-surface SEM micrograph of Al_2_O_3_ in different temperature: (**a**) 1000 °C, (**b**) 100 °C, (**c**) 1200 °C, (**d**) 1300 °C, (**e**) 1400 °C, (**f**) 1500 °C sintered by PPS.

The SEM images (Figure 10) obtained in secondary electron mode show that the samples’ characteristic areas of Al_2_O_3_ fractures of the samples sintered at different temperatures. The observations were carried out at fracture sites. SEM results revealed that the Al_2_O_3_ samples sintered at relatively low temperatures ranging from 1000 °C to 1300 °C are characterized by regular-shaped alumina particles with fractures (Figure 10a–d). In SEM images, the boundaries of all grains of the ceramic are visible, which confirms the intergranular fracturing and means that in this range of temperatures, the sintering process is not sufficient. This observation can be related to density measurements, which indicate that the lowest density is achieved during the sintering at temperatures 1000–1300 °C (Table 3).

**Figure 11:**
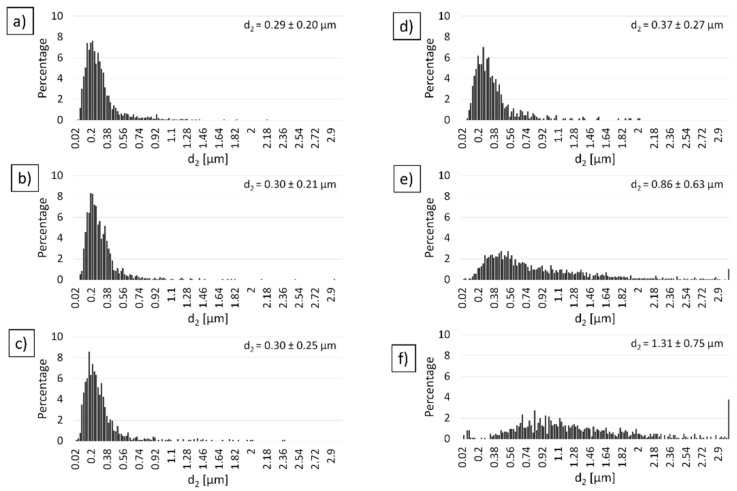
Histograms of the grain size distribution of Al_2_O_3_ depending on temperature: (**a**) 1000 °C, (**b**) 100 °C, (**c**) 1200 °C, (**d**) 1300 °C, (**e**) 1400 °C, (**f**) 1500 °C sintered by PPS method.

Moreover, the histograms shown in Figure 11a–d revealed a high percentage contribution of small (0.02–0.38 µm) grains in the composites sintered at temperatures 1000 °C–1300 °C. Small grains in the bulk ceramics confirm no sufficient sintering process in this range of temperatures. SEM observation of the fractures of the Al_2_O_3_ samples sintered at temperatures 1400 °C (Figure 10e) and 1500 °C (Figure 10f) revealed complete sintering of the alumina grains, which is confirmed by the high-density results (Table 3) and low percentage contribution of alumina grains with a size corresponding to the size of the initial powder (Figure 11e,f).

The next step was performed a stereological analysis based on the observation of fracture to determine the effect of the PPS process temperature on Al_2_O_3_ grain growth. The histograms of Al_2_O_3_ grain distribution in individual sinters (Figure 11) indicate that, along with the increasing temperature of the PPS process, the percentage amount of the larger particles in the microstructure increases.

Consideration of the results described above related to the Al_2_O_3_ powder compacted by PPS allows us to select the temperature of 1400 °C as the sintering temperature for the NiAl–Al_2_O_3_ composite powders. Although the density of compacted Al_2_O_3_ was only 97% at this temperature, the hardness was as high as for the sample compacted at 1500 °C, and the high K_IC_ value was obtained. Moreover, by choosing the temperature of 1400 °C decided that the ceramic grains did not grow for 1500 °C.

### 3.4. Characterization of NiAl–Al_2_O_3_ Powder Compacted by PPS

**Figure 12:**
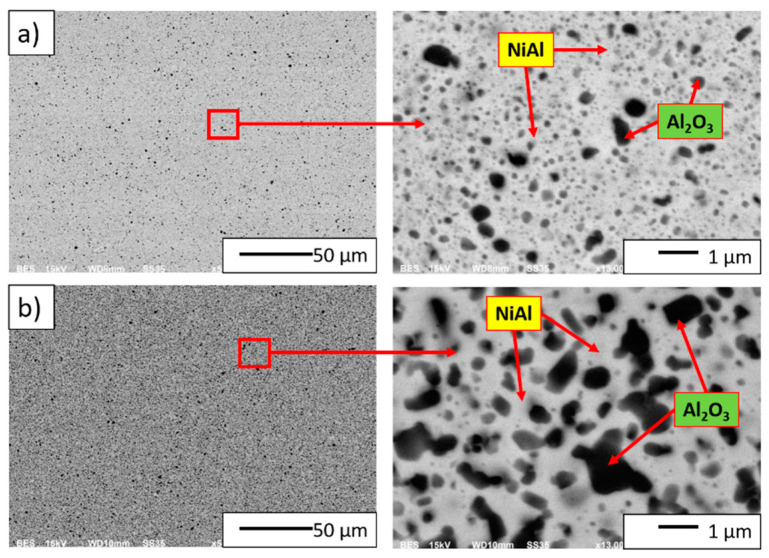
SEM images of microstructures after sintering by PPS at 1400 °C: (**a**) NiAl-10%Al_2_O_3_ sample, (**b**) NiAl-20%Al_2_O_3_ sample.

Figure 12 presents examples of SEM images of the NiAl-10%Al_2_O_3_ and NiAl-20%Al_2_O_3_ bulk specimens. The observation demonstrated that dark areas in the microstructure stand for the Al_2_O_3_, while the brightly grey areas correspond to the NiAl matrix. It was found that the surface of the polished samples was free of cracks and pores, which evidences the good quality of consolidation of specimens. SEM observations also showed that the samples had a homogeneous microstructure. Al_2_O_3_ inclusions are present in the NiAl matrix.

Based on the measurements from the pycnometer, it was found that a relative density of 97.9% characterized the samples containing 10 wt.% Al_2_O_3_ while the sample containing 20 wt.% Al_2_O_3_ was characterized by a relative density close to 100%. Based on density measurements by pycnometer, it was found that the relative density increases with the increase of Al_2_O_3_ content in the sinter.

**Figure 13:**
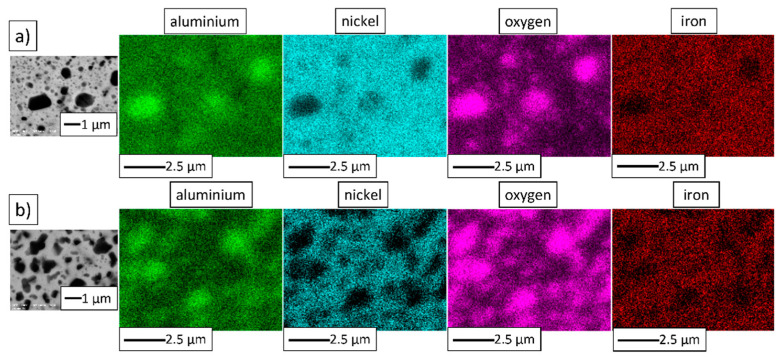
The specimens’ elemental distribution map after sintering by PPS at 1400 °C: (**a**) NiAl-10%Al_2_O_3_ sample, (**b**) NiAl-20%Al_2_O_3_ sample.

Subsequently, energy-dispersive X-ray spectroscopy (JEOL Ltd., Tokyo, Japan) was conducted to obtain the elemental distribution maps of the prepared NiAl-10%Al_2_O_3_ and NiAl-20%Al_2_O_3_ samples. The maps obtained are exhibited in Figure 13. The chemical element distribution maps revealed a non-uniform presence of aluminum, nickel, oxygen, and iron. Contamination of powders by iron from steel milling tools is generally recognized in mechanical alloying processes [28].

**Figure 14:**
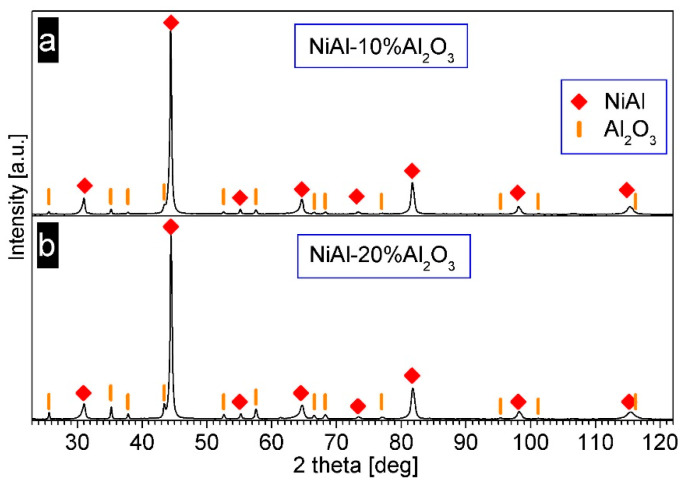
XRD patterns of the consolidated milling products: (**a**) NiAl-10%Al_2_O_3_ sample, (**b**) NiAl-20%Al_2_O_3_ sample.

Figure 14 presents the XRD patterns of the composite NiAl–Al_2_O_3_ powders after consolidation. Only the diffraction peaks of NiAl and Al_2_O_3_ are present, which shows that no phase changes occurred during the PPS process. The NiAl diffraction profiles’ widths are smaller than the NiAl peaks’ breadths in the powders. This sharpening of the peaks indicates grain growth in the NiAl phase during consolidation. The mean crystallite size estimated by the Williamson–Hall method was 112 nm and 123 nm for the sample containing 10% and 20% of Al_2_O_3,_ respectively. These values exceed 100 nm, so they are beyond the applicability limit of the Williamson–Hall method and may be affected by an error. However, it can be concluded that the NiAl phase has a submicrometer grain size.

For the hardness measurements of NiAl–Al_2_O_3_ composite samples sintered at 1400 °C, the hardness values achieved were naturally significantly lower than those for pure Al_2_O_3_ ceramics; however, for both the composite with lower (10 wt.% Al_2_O_3_) and higher (20 wt.% Al_2_O_3_) Al_2_O_3_ content, the HV values obtained were practically identical. They were equal to 5.8 ± 0.08 GPa and 5.8 ± 0.09 GPa, respectively. Interestingly, regarding the composite specimens in the research conducted by Marek Krasnowski et al. [32] on NiAl-B composites with nanocrystalline intermetallic matrix fabricated through mechanical alloying and consolidation, hardness results ranging from 10.3 GPa to 12.6 GPa were achieved. The lower hardness values observed in the present experiment are probably related to the growth of grains in the NiAl phase compared to the nanocrystalline grains observed in [32]. In the aforementioned study [32], the nanocrystalline structure was observed in the samples after consolidation. Another research on nanocrystalline NiAl carried out by the same research team showed that the presence of a nanocrystalline phase significantly exceeds the hardness compared to microcrystalline NiAl alloys. The hardness obtained for nanocrystalline NiAl amounted to 9.53 GPa [43]. According to literature data, the addition of Al_2_O_3_ to NiAl favorably affects the hardness of the material. Michalski et al. [55], in their work investigating the effect of Al_2_O_3_ on NiAl fabricated by the PPS method with the participation of the SHS reaction, reported a noticeable increase in hardness with increasing Al_2_O_3_ content. In the aforementioned work [55], the hardness of AlNi-Al_2_O_3_ composite with 38 vol.% Al_2_O_3_ was about 6.08 GPa, while the hardness of NiAl produced with the same process was equal to 4.22 GPa.

**Figure 15:**
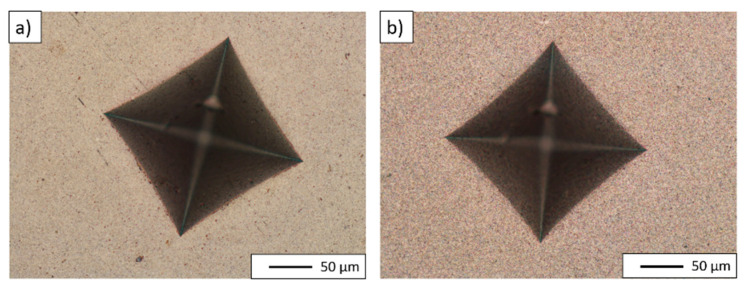
Vickers indenter traces in sintered samples: (**a**) NiAl-10%Al_2_O_3_ sample, (**b**) NiAl-20%Al_2_O_3_ sample.

Due to their high elasticity, the composite specimens did not fracture under the indentor impact, which can be seen in the exemplary indentations presented in Figure 15. Therefore, it was not possible to determine K_IC_ values for these specimens using the method applied in this research.

## 4. Conclusions

This work aimed to recognize the PPS method as a possible technique to produce bulk composite materials from NiAl–Al_2_O_3_ composite powder. The composite powder consisted of intermetallic NiAl, and Al_2_O_3_ was prepared by mechanical alloying. As an initial powder Ni, Al and Al_2_O_3_ were used.

As a preliminary study, the Al_2_O_3_ was consolidated by PPS and characterized. These experiments gave an important input in order to describe the sintering process in function of temperature. Mainly based on these results, the sintering temperature of the composite powders, which did not cause intensive grain growth, was estimated.

The results obtained revealed that the PPS method allows for the consolidation of ceramic and composite powders. In consolidated NiAl–Al_2_O_3_ powder, the bulk materials are generally set up in two phases: Al_2_O_3_ is located within the NiAl matrix. The intermetallic ceramic composites have relative densities: for composites with 10 wt.% Al_2_O_3_ 97.9% and samples containing 20 wt.% Al_2_O_3_ close to 100%. The hardness of both composites was equal to 5.8 GPa. Moreover, after PPS consolidation, NiAl–Al_2_O_3_ composites are characterized by high plasticity.

The results obtained are promising for further study of the consolidation of composite NiAl–Al_2_O_3_ powder with various initial contributions of ceramics (Al_2_O_3_) to prepare composite powder by mechanical alloying. Moreover, PPS could be applied to the consolidation of a mixture of intermetallic–ceramic composite powders with the addition of ceramics. This last option, when an amount of 50 or more % of Al_2_O_3_ will be mixed with NiAl–Al_2_O_3_ composite powder, can lead to the production of a new class of ceramic–intermetallic composites, with complex structure, i.e., Al_2_O_3_ ceramic matrix with areas of NiAl with fine particles of Al_2_O_3_ inside. Such composites should have attractive properties as well. The active contribution and synergistic effect of elements of the structure on the crack propagation and finally on improving the fracture toughness of the new composite are expected. Our work in this area is in progress.

## Figures and Tables

**Table 1:** The pulse plasma sintering (PPS) process parameters.

PPS Process Parameter	Al_2_O_3_ Samples	NiAl–Al_2_O_3_ Composite Samples
Stored Energy	4.06 ÷ 5.05 kJ	2.77 kJ
Voltage	5.2 ÷ 5.8 kV	4.3 kV
Electro-pulse repetition	1 ÷ 1.3 s	1.3 s
Heating rate	250 °C/min	250 °C/min
Sintering temperaturę	1000 °C, 1100 °C, 1200 °C, 1300 °C, 1400 °C, 1500 °C	1400 °C
Load	20–80 MPa	20–80 MPa
Sintering time	3 min	3 min

## Data Availability

Data sharing not applicable.
